# Challenges of making healthy lifestyle changes for families in Aotearoa/New Zealand

**DOI:** 10.1017/S1368980020003699

**Published:** 2021-05

**Authors:** Cervantée EK Wild, Ngauru T Rawiri, Esther J Willing, Paul L Hofman, Yvonne C Anderson

**Affiliations:** 1 Liggins Institute, University of Auckland, Auckland, New Zealand; 2 Department of Paediatrics: Child and Youth Health, University of Auckland, Auckland, New Zealand; 3 Tamariki Pakari Child Health and Wellbeing Trust, Taranaki, New Zealand; 4 Kōhatu – Centre for Hauora Māori, University of Otago, Dunedin, New Zealand; 5 Starship Children’s Hospital, Auckland District Health Board, Auckland, New Zealand; 6 Department of Paediatrics, Taranaki District Health Board, New Plymouth, New Zealand

**Keywords:** Paediatric obesity, Healthy lifestyle, Intervention, Obesogenic environment, Qualitative, Community nutrition

## Abstract

**Objective:**

The objective of the current study was to identify challenges of making and sustaining healthy lifestyle changes for families with children/adolescents affected by obesity, who were referred to a multicomponent healthy lifestyle assessment and intervention programme in Aotearoa/New Zealand (NZ).

**Design:**

Secondary qualitative analysis of semi-structured interviews.

**Setting:**

Taranaki region of Aotearoa/NZ.

**Participants:**

Thirty-eight interviews with parents/caregivers (*n* 42) of children/adolescents who had previously been referred to a family-focused multidisciplinary programme for childhood obesity intervention, who identified challenges of making healthy lifestyle changes. Participants had varying levels of engagement, including those who declined contact after their referral.

**Results:**

Participant-identified challenges included financial cost, impact of the food environment, time pressures, stress, maintaining consistency across households, independence in adolescence, concern for mental health and frustration when not seeing changes in weight status.

**Conclusions:**

Participants recognised a range of factors that contributed towards their ability to make and sustain change, including factors at the wider socio-environmental level beyond their immediate control. Even with the support of a multidisciplinary healthy lifestyle programme, participants found it difficult to make sustained changes within an obesogenic environment. Healthy lifestyle intervention programmes and families’ abilities to make and sustain changes require alignment of prevention efforts, focusing on policy changes to improve the food environment and eliminate structural inequities.

An estimated 11 % of children aged 2–14 years in Aotearoa/New Zealand (henceforth referred to as New Zealand (NZ)) are affected by obesity, and a further 20 % of children are classified as overweight^(1)^. Additionally, children living in the most deprived areas are twice as likely to experience obesity than those living in the least deprived areas^(1)^. In addition to population-level prevention efforts, it is important that children and youth experiencing weight issues are offered support to make healthy lifestyle changes; international recommendations for the management of childhood obesity include family-based healthy lifestyle programmes that incorporate nutrition, physical activity and psychosocial components^(2,3)^. NZ children with obesity show high rates of weight-related co-morbidities, as well as suboptimal dietary behaviours and low levels of physical activity, irrespective of ethnicity^(4–6)^. At a national level, only 50 % of NZ children (2–14 years) meet the Ministry of Health’s fruit and vegetable intake guidelines^(7)^. Additionally, 89 % of children (6 months–14 years) do not meet screen time recommendations for their age group, and 23 % (0–14 years) have insufficient sleep^(7)^. However, there is currently fragmented provision of multicomponent healthy lifestyle programmes for children and adolescents in NZ, which means access to these services remains limited.

Implementing and sustaining healthy lifestyle changes can be challenging due to a wide range of individual, social and environmental factors – a 2017 review of barriers and enablers to healthy nutrition, physical activity, sedentary activity and sleep habits in adolescents found that most studies had focused on individual and interpersonal barriers and enablers, and few studies have explored environmental and policy-level influences^(8)^. Kebbe and colleagues’ subsequent multi-centre qualitative study in Canada of barriers and enablers of healthy lifestyle behaviours in adolescents with obesity found that physical and mental health, self-efficacy (in terms of self-regulation, controllability and competence beliefs), social relationships and accessibility of opportunities for lifestyle change all affected adolescents’ abilities to make changes as barriers and/or enablers^(9)^. This reflected findings from an interview-based evaluation of barriers and facilitators of goal achievement in the Parenting Eating and Activity for Child Health trial for children experiencing overweight in Australia, which highlighted the relevance of factors external to a support programme and beyond the parental locus of control^(10)^.

Previous research in NZ into the challenges of making healthy lifestyle changes has largely focused on the cost of eating healthily in an obesogenic (obesity-promoting) environment^(11,12)^. A qualitative study of the facilitators and barriers to achieving a healthy weight in children among focus groups with Māori (the Indigenous people of NZ) parents and caregivers demonstrated that a key barrier to making healthy food choices was cost, but this was closely related to lack of time, the number of people to feed and individual preferences^(13)^. In addition, food provisioning decisions were complex and involved weighing up the relative importance of ensuring both child health and happiness^(13)^. While multidisciplinary interventions can provide support for children with obesity and their families, there are potentially enduring influences on nutrition and physical activity outside of the family control^(10)^. It is important that families are able to sustain healthy lifestyle changes in order to achieve reduction in weight status over time and address weight-related co-morbidities.

One family-based multidisciplinary programme focused on supporting families to make healthy lifestyle changes in NZ is Whānau Pakari, which means ‘self-assured whānau (families) who are fully active’. Whānau Pakari eligibility criteria are children and adolescents aged 5–16 years with a BMI ≥ 98th centile, or those > 91st centile with weight-related co-morbidities. The programme is more accessible than previous models^(14)^, with a ‘demedicalised’ approach to addressing childhood obesity. Medical assessments occur in the home, which is more acceptable to the community. Focus groups with past Whānau Pakari participants showed that participants and their caregivers valued the sense of connectedness, knowledge sharing, the experience of the collective journey and the respectful, non-judgemental environment in the family-based programme^(15)^. Whānau Pakari has demonstrated effectiveness in a randomised clinical trial, which showed improvements in physical activity, psychological outcomes and BMI SD score at 12 months^(16)^. Whilst the BMI SD score reductions did not persist at 24 months, reductions in sweet drink intake, increases in water intake and improvements in cardiovascular fitness and health-related quality of life were present^(16,17)^, as well as qualitative evidence of a range of health and well-being benefits^(15,18)^.

This paper presents a secondary analysis of data from the Whānau Pakari barriers and facilitators study^(19)^. This involved understanding the factors influencing engagement and retention in Whānau Pakari through a survey and in-depth, semi-structured interviews with past participants and their families who had been referred to the programme^(18)^, given attendance at programme sessions had been previously associated with improved health outcomes^(16,17)^. The content of the interviews was broad, ranging from participants’ experiences in the Whānau Pakari programme itself to wider experiences in the healthcare system. Overall, participant experiences with Whānau Pakari have been explored previously^(19)^. While it was not solicited by the interviewers, over half of participants also volunteered information about the challenges of healthy lifestyle change. This was not a focus of the interview and the information was not requested by the interviewers, but it was clearly an important topic for participants and part of their experience of engaging in a healthy lifestyle programme. Although it was not part of the primary analysis of the interview data, it was important that participant voice was reflected in the wider research project. The research team considered the challenges discussed by participants to be an element to consider in the context of addressing childhood obesity in NZ. The objective of this secondary analysis was therefore to identify the challenges of making and sustaining healthy lifestyle changes for families with children and adolescents who were referred to a multidisciplinary healthy lifestyle programme.

## Methods

### Programme context

Whānau Pakari is an assessment and intervention programme involving weekly group sessions delivered by a multidisciplinary team, including a physical activity coordinator, dietitian and psychologist^(16,20)^. The sessions incorporated family physical activity sessions, including a variety of sports and games to engage participants’ interests; psychology sessions, including discussion of topics such as self-esteem and how to maintain healthy lifestyle changes; and dietary sessions, including portion sizes, the concept of healthy food, virtual supermarket tours and cooking sessions^(20)^. These sessions took place in a community-based sporting venue, and the assessments took place in participant homes in order to minimise travel barriers.

### Whānau Pakari barriers and facilitators study

The methodology for the current study has been published previously^(19)^. Briefly, in-depth, home-based interviews were undertaken with parents, caregivers and past participants of Whānau Pakari who had engaged with Whānau Pakari to varying degrees, from those with high attendance to those who were referred to the service and declined input, in order to ascertain how engagement could be improved. Interviews were chosen over focus groups, due to the sensitivity of the topic, and with the anticipation that participants who were reluctant to engage with the group setting of the programme would prefer to participate in interview-based research rather than focus group research.

Eligible participants were parents and caregivers of children and adolescents (and children themselves if over 11 years) who had been referred to the programme from January 2012 to January 2017. Recruitment was by text message and telephone call and included equal numbers of Māori and non-Māori families. The interview schedule was developed based on previous literature^(18,21)^ and consultation with the Whānau Pakari programme delivery team, who provided anecdotal evidence from participant assessments. Participants were asked about their experiences in the Whānau Pakari programme and about what affected their ability to engage, in order to ascertain factors which affected engagement (see online supplementary material, Supplemental File 1).

‘Community-Up’ research principles were utilised to develop rapport with the participants^(22,23)^, which allowed for rich data collection. C.E.K.W. and N.T.R. conducted the interviews jointly, and N.T.R. led the interviews with Māori families when appropriate. Conducting the interviews jointly as a team with a Māori interviewer alongside a Pākehā (non-Māori) interviewer facilitated the relationship-building process with participants, particularly with Māori whānau (families). Participant ethnicity for the parent/caregiver and child was confirmed during the interview using the NZ Census 2018 ethnicity question^(24)^. A koha (gift, donation or contribution) was offered in reciprocity for participants’ time. The interviews were audio recorded and independently transcribed. No formal member-checking process to assess credibility of results was undertaken; however, transcripts were returned to participants for accuracy and acceptability checks, and a summary feedback video was provided, which was indicated by participants as preferable over a feedback hui (meeting) or written report. Further details on the interview procedure can be found in the consolidated criteria for reporting qualitative research checklist in online supplementary material, Supplemental File 2.

### Secondary analysis

Interview transcripts were inductively coded and analysed in MAXQDA software using reflexive thematic analysis^(25)^. The code ‘Challenges of Healthy Lifestyle Change’ identified in the original analysis was used to screen the interviews for relevant material. Participant transcripts were included in the analysis if they had discussed the challenges of healthy lifestyle change in their interview (*n* 38 of the original sixty-four interviews). C.E.K.W. conducted the initial analysis with supervision from E.J.W. The interview data were coded to identify new concepts which emerged as challenges evolved. New codes were added to the coding matrix as they emerged. The final coding matrix was peer reviewed by the research team to assess reliability and consensus. The codes were then amalgamated into the final themes, which were compared across engagement level and ethnicity to identify any potential differences. The research team collaborated to finalise the themes, with previously agreed respectful parameters allowing the authors to debate, challenge and refine interpretations of the data. Given this was a collaboration between Maori and non-Maori researchers, there were explicit agreements in place from the beginning of the study, where control over the analysis and interpretation of Maori data was held by Maori research team members^(26)^. It was agreed to apply the ‘Give-Way’ rule throughout the wider study if there was disagreement over the interpretation of the data concerning Māori participants^(26–28)^, whereby any ﬁnal decision involving interpretation of Māori participants’ experiences would pass to a Māori researcher.

## Results

The demographic characteristics of the thirty-eight participants included in the secondary analysis are presented in Table 1. Seventeen interviews were with families with Māori children (Māori or non-Māori parent/caregiver). No interviews included in this secondary analysis included children or adolescents as participants, as the topic of implementing healthy lifestyle changes was not raised by these participants.

**Table 1:** Interview participant demographics (parents or caregivers of children and adolescents referred to the Whānau Pakari service) included in secondary analysis*

	*n*	%
Interview participants	42	
Female	36	86
Ethnicity†		
Māori	14	33
NZ European	27	64
Other European	1	2
Level of engagement‡		
Attended ≥70 % of programme sessions	11	29
Attended <30 % of programme sessions	11	29
Had one assessment, then discontinued with the programme	4	11
Referred, but chose not to engage	12	32

* *n* 38 interviews included in secondary analysis.

† Total ethnicity output (more than one ethnicity selected, total adds to >100)^(24)^.

‡ Refers to number and percentage of interviews. Four interviews involved two family members.

Participants described a range of factors that influenced their ability to both implement and maintain positive healthy lifestyle changes, identified and supported by example participant quotations in Table 2. These key findings were consistent across engagement level and ethnicity. Overall, participants had a sense that a wide range of factors contributed towards someone experiencing weight issues, and therefore a range of factors contributed towards their ability to make healthy lifestyle changes:

‘You’ve got the economic one, you’ve got the social one, you’ve got the individual one, you’ve got the monetary one, and all those factors contribute into why someone is overweight.’ – mother, Māori

**Table 2:** Participant-identified challenges of healthy lifestyle change

Factors	Quotation to illustrate identified challenges
Financial cost	‘Fruit and veggies, they encourage you to eat them, but I can spend $40 odd, $50 just on fruit and veggies a week out of my groceries, and then, you know, you’ve still got your meat and everything else on that it’s not cheap to live (…) there’s people worse off than me that really struggle. So you look at your food and then your health system, like, doctors and then your medicines, it all adds up.’ – *mother, New Zealand European*
Food environment	‘…you sit there and you’re not hungry, after about five minutes of ads about food and junk food then you think ‘oh I might go have a biscuit or a cup of tea’ – *mother, Māori*
Time pressures	‘I mean, when you’re in a busy life you haven’t got time to go reading everything on the box in the supermarket have you?’ – *mother, New Zealand European*
Stress	‘I always have to watch my weight and I am trying to be a mother and I might have to go to work as well and I’m trying to figure what everybody is going to eat every day and it’s exhausting. How do I keep everybody on track and not fall over?’ – *mother, Māori*
Consistency across households	‘I had to knuckle down on my partner with my daughter because he’d be like ‘I’m allowed to give her a treat’ and I’m like ‘yeah, and then she goes down to Nanny’s and Nanny has made her cupcakes and then she goes over to Koro’s and he’s done all these things, and her aunties will give her all these things’. It all adds up to lots of sugar.’ – *mother, Māori*
Independence in adolescence	‘So when I controlled everything, she was younger, we got her weight down (…) I could control it, but as she’s got to teenage years, we have our license now, we have a job. We can’t control it. We’re our own identity so now she’s her own, she is her own weight and I can’t do anything about it.’ – *mother, New Zealand European*
Concern for mental health	‘I just don’t want him to have any issues about it. We try our best to make sure he eats good stuff.’ – *mother, New Zealand European (child is Māori)*
Frustration when not seeing change	‘(DAUGHTER)’s always been big. She’s still a big girl now. But whatever she tries to do, nothing works. She would eat nothing, she could just drink water, and still gain weight. She’s just one of those people. The more exercise she does, it does nothing.’ – *mother, New Zealand European*

### Financial cost of healthy eating

Participants stated that the cost of healthy food was off-putting, impacting their ability to make healthier choices. This cost was an extra burden on the family when they did choose to purchase it, especially with larger families. Buying in bulk was cheaper but put pressure on families due to the upfront cost.

‘So when you think of meat, fruit, vegetables, it is quite a costly thing, and we lived in a house of essentially nine people, so it was a massive meal to make and money was extremely tight because there was seven kids and, you know, two adults, so it was quite hectic.’ – mother, Māori

‘Food’s not cheap and especially when you take them off formula and put them on normal cow’s milk […] we go through 6+ litres of milk a week. I buy the 3 litres ’cause it works out a little bit cheaper, but 3 litres is still $5 something so, you know, in your groceries so there’s $10-$15 just for milk.’ – mother, New Zealand European

Many families on one income struggled to make healthier choices, and due to the cost of changing to healthy food for the whole family, some families made the choice to prepare healthy meals for the child who needed it most.

‘The cost of changing the food and, you know, for one particular person when you’ve got like two or three kids and stuff like that does make it a lot. When you’ve got one child it’s not too bad if you’ve got two incomes, but when you’ve got, say like a solo parent and you’ve got three or four kids, trying to give that other kid extra decent stuff that they need. I mean, they all should have it, but, you know, when you’re doing one you can afford it, but when you’ve got two or three that you are trying to get to eat healthy as well [it] does put a big toll on it. I mean, we tried it, I tried it quite a while with [SON] and it was just like, it was getting costly and then I was only part-time working then and it was a big struggle.’ – mother, Māori

In addition, for families struggling with ongoing food insecurity, it was more important that the family had some food than no food:

‘We went to a family group conference […] she said oh they didn’t have very good lunches, and I said, “I beg your pardon?” Because I had been there a lot of times when she’d made their lunches and taken them up to school. On a pay day she would go to the supermarket in the morning and then take their lunches to them so that she had, they had lunches, and if they didn’t have lunches, she didn’t send them to school.’ – grandmother, New Zealand European (grandchildren are Māori)

This participant stated, ‘they may not have been the healthiest of lunches, but there was plenty of it’, demonstrating the variation in identified priorities relating to food for families.

### Effect of the food environment

The food environment contributed towards food provisioning decisions and physical activity, such as the types of food outlets available and food advertising. Another participant recognised the effects of food advertising on her family’s ability to make healthy choices (Table 2). One participant stated that the introduction of a fruit shop to their small town (population of <2000 people) had changed residents’ eating habits:

‘…until the past two months we couldn’t afford the fruit and veggies. Now we’ve got a fruit shop in town that sells seconds and local produce, you know […] So people in this town, that fruit shop has given hundreds of people on no incomes, you know, it changed our diet and it’s making a difference.’ – grandfather, New Zealand European (grandchildren are Māori)

Participants recognised that a change in environment affected their abilities to make healthy lifestyle changes:

‘I visit my country and we eat more vegetables and more walk because my mum not drive, and she’s just skinny, skinny and skinny. When we come back she start again [to] eat.’ – mother, Other European

### Time available to make healthy meals

Many families stated it was the time cost involved that was a barrier to eating healthily – this included planning, buying, preparing, cooking and cleaning up. This was often difficult or unrealistic for families working long hours or with only one parent available to prepare meals.

‘And with how our lifestyles worked, it just wasn’t realistic […] by the time we all get home the last thing any of us wanted to do was dick around with a long-term meal. They just wanted food on the table ’cause they’re starving and I didn’t want to do anything so, you know, we do fall back on things like macaroni cheese and sausages and things like that because it was easy.’ – mother, New Zealand European

### Stress of implementing healthy lifestyle changes

Similarly, the stress of trying to implement healthy lifestyle changes was often perceived to be too much for families depending on the time available, work hours, family size and competing priorities.

‘It’s small changes in small periods of time because otherwise you have rip roaring arguments at home and children are detoxing off sugar over here and mum’s over in the corner wanting a drink going ‘oh my God’, feeling, the screaming, the fighting over it. It just wasn’t worth it.’ – mother, New Zealand European

Making healthy lifestyle changes around nutrition was also difficult compared with other lifestyle factors such as smoking, due to the necessity of food in everyday life:

‘It’s like people who smoke and people who drink - they know that they shouldn’t, but they still do. *Laughing.* The thing is, you can’t just quit food. Oh, I’ll just quit eating. *Laughing.* I’ll just quit, I’ll just go cold turkey and I won’t eat any more ever. It doesn’t work.’ – mother, Māori

In many cases, parents and caregivers simply wanted to keep their children happy, which often equated to them being full. Some participants spoke of how difficult it was to consistently make healthy lifestyle changes when families were dealing with other complex issues:

Participant 1: ‘It did affect our kids though, I must notice, when that happened [imprisonment]. Our kids started getting judged from our actions, aye.’ – father, Māori

Participant 2: ‘Yeah, that’s when I let them eat whatever they wanted, gave them whatever they wanted. They were crying, “I was missing Dad”, so here eat whatever you want. Lost control of them.’ – mother, Māori

### Consistency across family

It was difficult to maintain consistent healthy lifestyle changes across families with parents living in separate households.

‘[DAUGHTER, 17], at home her diet, we didn’t have that stuff in the house so her diet, she ate the same as what the other two girls ate. The other two girls weren’t overweight. It was when she went to the other house, there was access to coke [Coca-Cola] and so she binged on it.’ – mother, New Zealand European

Participants also expressed frustration when other family members fed their children what they wanted rather than trying to maintain changes.

‘Nannies and Grandads man – they’re shocking. Like, I tried to get to my parents onboard and I’m like ‘look, we need to watch what she eats, you know, don’t give her any lollies and all this stuff and fizzies’ and oh yeah, no. ‘No, we’re grandparents, we’re allowed to do that’. Well, you’re affecting her then, aren’t they? […] They’re like ‘they’re my moko [grandchildren], they’re alright’. […] When everybody else gives them treats, aye, it all adds up.’ – mother, Māori

### Balancing healthy lifestyle changes with concern for mental health

There was concern from some families about the risk of stigmatising children based on their size, and strong concerns about child and adolescent mental health. For some families, this was considered more important than implementing healthy lifestyle changes relating to weight. For other families trying to make healthy lifestyle changes relating to weight, this was a delicate balance between encouraging healthy choices and protecting self-esteem.

‘Having had conversations with friends who have had daughters who have suffered from eating disorders, and having had a sister who did, my kind of feeling is that the best approach is the fairly low-key relaxed. Just let her kind of figure it out, try and be there, not ask her lots of questions and not do the whole, “what are you eating?” “How long did you exercise for?” I just think you just have to keep it a little bit light and yeah, trust that she’ll figure it out.’ – mother, New Zealand European

### Independence in adolescence

It was difficult for parents/caregivers to help their older children to make or maintain changes as they moved into adolescence. Many adolescents started working and gained more financial independence. Parents expressed how difficult it was to continue to support their teenage children to make healthy lifestyle changes (Table 2). The introduction to alcohol in adolescence was another challenge due to the social pressure to drink alcohol with their peers:

‘Now they all drink so it’s like “grrr, do you realise how much sugar those fucking crap drinks have in them [DAUGHTER]?” Bloody RTD [ready to drink alcohol mixes] thingies, KGBs [alcohol brand]…’ – mother, New Zealand European (child is Māori)

### Frustration when not seeing change/maintaining motivation

A key challenge for maintaining healthy lifestyle changes was frustration when participants did not see changes in their health or body weight. Many participants stated how difficult it was to continue with the lifestyle changes they had made because it did not seem to make a difference. This was discouraging for many families who may have made positive healthy lifestyle changes but felt like they had still ‘failed’. One participant speculated that this was why many people did not continue with their lifestyle:

‘It’s a mystery to me why he didn’t [lose weight] … All I can think of is that I feel like I’m still big, I’m still, overweight, and he’s still overweight […] It’s reflecting on me – “look, I’m still the same, I’m not doing it properly, people are going to say to me, ‘you’re not doing it properly’”.’ – mother, New Zealand European

Making lifestyle changes in the hope of losing weight was considered a futile endeavour by some participants, which was a key barrier for maintaining healthy lifestyle changes (Table 2).

**Fig. 1:**
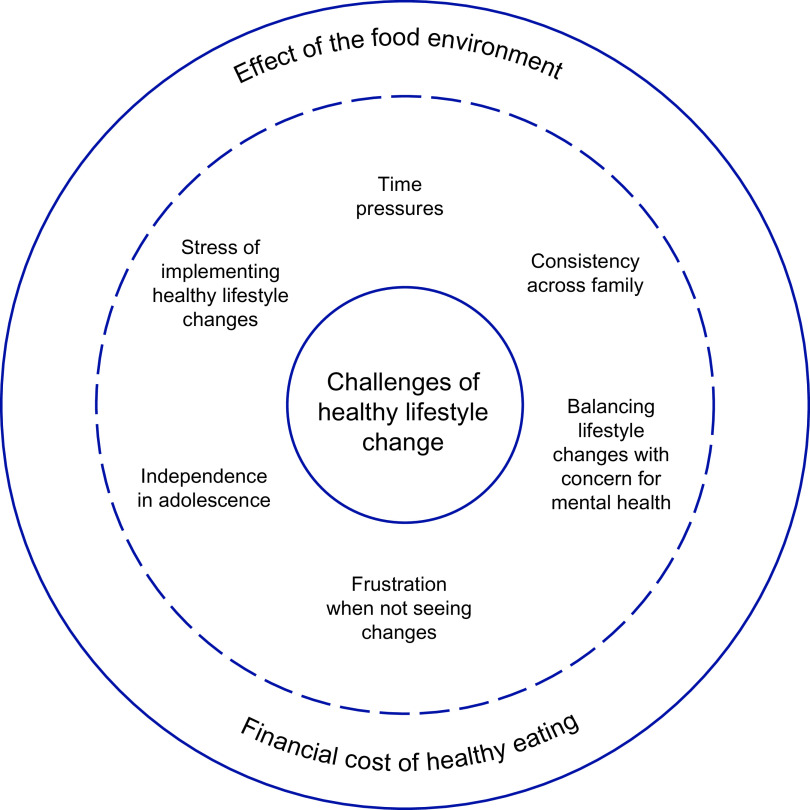
Participant-identified challenges of implementing and sustaining healthy lifestyle changes: financial cost of healthy eating, the effect of the food environment, time pressures, the stress of implementing healthy lifestyle changes, maintaining consistency across family, independence in adolescence, balancing lifestyle changes with concern for mental health and frustration when not seeing changes. The financial cost of healthy eating and effect of the food environment overlies the remaining factors, exacerbating their effects

These themes are depicted in Fig. 1, which identifies the financial cost of healthy eating and effect of the food environment as overlying themes which exacerbated the effects of the remaining six factors.

## Discussion

The current study shows that families in NZ face a range of challenges when attempting to make healthy lifestyle changes in current environments. Participant-identified challenges were the financial and time cost of eating healthily, the stress of making healthy lifestyle changes, the effect of the food environment, maintaining changes across split households, concern for child mental health, increased independence in adolescence and frustration when not seeing changes. Our results suggest that implementing healthy lifestyle changes is challenging even with the support of a healthy lifestyle intervention due to a range of external socio-environmental factors. The Whānau Pakari cohort had 49 % of participants over split households^(4)^, highlighting the challenge of achieving healthy lifestyle change across multiple home environments in this group.

Previous research in NZ has shown that food provisioning decisions are affected by a wide range of factors such as cost, tiredness, stress, lack of help, time, food preferences and access to food, many of which can be mitigated by the economic determinants of food insecurity^(13)^. Our study supports this finding and similarly identifies the multiple facets of the cost of healthy eating, which includes not only the financial cost of purchasing healthy food^(11,12)^ but also the time cost and additional stress of preparing healthy meals^(13)^. The range of stressors on families is mentally taxing and families may be less able to make healthy lifestyle changes when dealing with multiple other concerns, especially in the context of the obesogenic environment and entrenched socio-economic inequities.

Our study identified independence in adolescence as being a key challenge of sustaining healthy lifestyle changes, in terms of both access to resources which increase autonomy such as a part-time job or driver’s license and the introduction of alcohol consumption as an additional source of energy. The Parenting Eating and Activity for Child Health trial evaluation previously identified peer pressure as a major barrier to achieving healthy lifestyle goals in children aged 5–10 years^(10)^. This was also confirmed as a relevant factor in Kebbe and colleagues’ study of barriers and enablers to adopting healthy lifestyle behaviours for adolescents, which highlighted the role of peer pressure to conform to social expectations with regard to eating behaviours^(9)^. Future interventions could consider an adolescent-specific programme which deals with the unique challenges of making healthy lifestyle changes in adolescence, alongside restriction on the marketing of unhealthy foods and beverages to children and adolescents.

Family- and community-based multidisciplinary interventions are needed given the prevalence of weight-related co-morbidities in NZ children^(4)^ and are one of six key recommendations by the WHO’s Report of the Commission on Ending Childhood Obesity^(29)^. Multidisciplinary programmes that are accessible and acceptable to the community are critical for addressing weight issues. However, the identification by participants of the clear effect of the food environment on peoples’ ability to sustain healthy lifestyle changes further reinforces the need to make healthy choices easier through addressing food environments at a policy level^(30)^, as well as addressing income and socio-economic inequity^(31)^. Therefore, community-based interventions need to be provided within the context of wider preventative efforts^(32)^.

While findings were consistent across Māori and non-Māori families in terms of identified challenges, it is likely that socio-economic inequities contribute towards the ability of participants to sustain healthy lifestyle changes. In NZ, socio-economic status and ethnicity are highly correlated characteristics; more than 24 % of Māori live in the most deprived areas of NZ, compared with 7 % of non-Māori^(33)^. This patterning is evident internationally, and socio-economic deprivation, along with racial discrimination, has been identified as a key determinant of inequities in healthcare access and resource for Indigenous peoples^(34)^. It is critical that achieving health equity is prioritised in service provision in order to enhance outcomes for Indigenous peoples, especially in complex health areas like healthy lifestyle change. The effectiveness of intervention programmes and families’ abilities to sustain healthy lifestyle changes are therefore likely to be enhanced by policies which focus on improving the food environment and decreasing the cost of healthy food. Given children and adolescents live in families, and families live in communities, it remains difficult for persistent healthy lifestyle change to be achieved when the surrounding environment remains obesogenic.

The frustration and shame experienced by families if they did not see weight changes despite the adoption of many healthy habits reinforces the need for a range of health and well-being goals and indicators of success in multidisciplinary interventions. The utility of BMI as a sole measure of intervention success has previously been questioned^(35)^, particularly as a relevant measure for Indigenous groups^(36)^. A lack of reduction in BMI should not be equated with failing to adopt healthy lifestyle changes, and an over-focus on weight in childhood obesity interventions is a missed opportunity for many families who have made positive dietary and physical activity changes^(35)^. It is acknowledged that improvements in weight status are key in addressing weight-related co-morbidities long-term.

The Whānau Pakari programme takes a non-stigmatising, non-judgemental approach, with a focus on healthy lifestyle change rather than using terms such as weight loss, diet or obesity. However, participants are still inundated with messages from wider society that reinforce the idea that ‘success’ is solely weight loss over a set period of time, while trying to make changes in an obesogenic environment, which may be discouraging for participants. In addition to policies which focus on reducing the effect of the obesogenic environment, a societal shift to address the stigma associated with weight is also required. Future programmes should include a range of health-related indicators and outcomes and should prioritise the voices, needs and preferences of children and their families in terms of the feedback of healthy lifestyle messages, measures and progress. This would allow for more tailored messaging and feedback that is deemed relevant for each family’s preferences and concerns.

A strength of the current study is that the data are likely to be reliable and not influenced by social desirability bias, as it was freely offered by participants and not a focus of the interview. Although participants were offered their transcripts to check for accuracy and acceptability in the original study, no member-checking process was used to assess the credibility of results with participants. A limitation of the secondary analysis is that it was not possible to ask further explanatory questions that might have further clarified the data. For example, many of the participant accounts focused on challenges around healthy food, and notably missing from the data is discussion of the challenges of making changes in physical activity, sedentary activity and sleep hygiene behaviour. Also missing are factors that enable families to make healthy lifestyle changes, in addition to the barriers. Given the low numbers of NZ children meeting national recommendations in these areas^(7,37)^, this is an important element of understanding the effectiveness of interventions in NZ. Parental and caregiver identification of adolescence as a difficult period for healthy lifestyle change reflects the need to prioritise child and adolescent voice, which the current study was unable to do, and should be a focus of future research in this area. Previous qualitative research has recommended that lifestyle interventions for adolescents should emphasise a broader range of outcomes than weight, particularly focusing on mental health as an outcome^(9)^.

## Conclusion

In conclusion, families in NZ face a range of challenges at both the individual and interpersonal to socio-environmental levels that impede their ability to make and sustain healthy lifestyle changes, even with the support of a healthy lifestyle programme. The effectiveness of intervention programmes in a real-world setting and the ability of families to achieve persistent healthy lifestyle changes would be enhanced by aligned, coordinated policies which focus on improving the food environment in order to make it easier for families to make persistent healthy lifestyle changes. Sustained healthy lifestyle change will require substantial action on both the obesogenic environment and other social determinants of health.

## Supplementary material

For supplementary material accompanying this paper visit https://doi.org/10.1017/S1368980020003699. click here to view supplementary material

## References

[1] Ministry of Health New Zealand (2019) Tier 1 statistics 2018/2019: New Zealand Health Survey. https://minhealthnz.shinyapps.io/nz-health-survey-2018-19-annual-data-explorer/_w_104ecbf8/#!/home (accessed February 2020).

[2] World Health Organization (2016) Report of the Commission on Ending Childhood Obesity. Geneva, Switzerland: World Health Organization.

[3] Ho M , Garnett SP , Baur L et al. (2012) Effectiveness of lifestyle interventions in child obesity: systematic review with meta-analysis. Pediatrics 130, e1647–1671. 23166346 10.1542/peds.2012-1176

[4] Anderson YC, Wynter LE, Treves KF et al. (2016) Prevalence of comorbidities in obese New Zealand children and adolescents at enrolment in a community-based obesity programme. J Paediatr Child Health 52, 1099–1105. 27634284 10.1111/jpc.13315

[5] Anderson YC, Wynter LE, Butler MS et al. (2016) Dietary intake and eating behaviours of obese New Zealand children and adolescents enrolled in a community-based intervention programme. PLoS ONE 11, e0166996. 27880804 10.1371/journal.pone.0166996 PMC5120841

[6] Anderson YC, Wynter LE, Grant CC et al. (2017) Physical activity is low in obese New Zealand children and adolescents. Sci Rep 7, 41822. 28157185 10.1038/srep41822 PMC5291106

[7] Ministry of Health NZ (2017) Tier 1 statistics 2016/17: New Zealand Health Survey. https://minhealthnz.shinyapps.io/nz-health-survey-2016-17-tier-1/ (accessed March 2019).

[8] Kebbe M , Damanhoury S , Browne N et al. (2017) Barriers to and enablers of healthy lifestyle behaviours in adolescents with obesity: a scoping review and stakeholder consultation. Obes Rev: Offic J Int Assoc Study Obes 18, 1439–1453. 10.1111/obr.12602 28925065

[9] Kebbe M , Perez A , Buchholz A et al. (2018) Barriers and enablers for adopting lifestyle behavior changes in adolescents with obesity: a multi-centre, qualitative study. PLoS One 13, e0209219. 30562377 10.1371/journal.pone.0209219 PMC6298663

[10] Perry RA , Daniels LA , Bell L et al. (2017) Facilitators and barriers to the achievement of healthy lifestyle goals: qualitative findings from Australian parents enrolled in the PEACH Child Weight Management Program. J Nutr Educ Behav 49, 43–52. 27780669 10.1016/j.jneb.2016.08.018

[11] Wang J , Williams M , Rush E et al. (2010) Mapping the availability and accessibility of healthy food in rural and urban New Zealand-Te Wai o Rona: diabetes Prevention Strategy. Public Health Nutr 13, 1049–1055. 19781125 10.1017/S1368980009991595

[12] Mackay S , Vandevijvere S , Xie P et al. (2017) Paying for convenience: comparing the cost of takeaway meals with their healthier home-cooked counterparts in New Zealand. Public Health Nutr 20, 2269–2276. 28625211 10.1017/S1368980017000805 PMC10261667

[13] Glover M , Wong SF , Taylor RW et al. (2019) The complexity of food provisioning decisions by māori caregivers to ensure the happiness and health of their children. Nutrients 11, 994. 10.3390/nu11050994 PMC6566933 31052332

[14] Anderson YC, Taylor GM, Grant CC et al. (2015) The green prescription active families programme in Taranaki, New Zealand 2007–2009: did it reach children in need? J Prim Health Care 7, 192–197. 26437042

[15] Anderson Y (2018) Whānau Pakari: a multi-disciplinary intervention for children and adolescents with weight issues. PhD Thesis, University of Auckland. 10.1111/ijpo.12693 32959996

[16] Anderson Y, Wynter L, Grant C et al. (2017) A novel home-based intervention for child and adolescent obesity: the results of the Whānau Pakari randomized controlled trial. Obesity 25, 1965–1973. 29049868 10.1002/oby.21967

[17] Anderson Y, Wynter L, O’Sullivan N et al. (2020) Two-year outcomes of Whānau Pakari, a multi-disciplinary assessment and intervention for children and adolescents with weight issues: a randomised clinical trial. Pediatr Obes, e12693. 32959996 10.1111/ijpo.12693

[18] Wild CEK, O’Sullivan NA, Lee AC et al. (2020) Survey of barriers and facilitators to engagement in a multidisciplinary healthy lifestyles program for children. J Nutr Educ Behav 52, 528–534. 31780274 10.1016/j.jneb.2019.10.010

[19] Wild CE, Rawiri N, Willing EJ et al. (2020) Determining barriers and facilitators to engagement for families in a family-based multicomponent healthy lifestyles intervention for children and adolescents. BMJ Open 10, e037152. 10.1136/bmjopen-2020-037152 PMC7478027 32895279

[20] Anderson YC, Wynter LE, Moller KR et al. (2015) The effect of a multi-disciplinary obesity intervention compared to usual practice in those ready to make lifestyle changes: design and rationale of Whanau Pakari. BMC Obes 2, 41. 26464806 10.1186/s40608-015-0068-y PMC4599755

[21] Kelleher E , Davoren MP , Harrington JM et al. (2017) Barriers and facilitators to initial and continued attendance at community-based lifestyle programmes among families of overweight and obese children: a systematic review. Obes Rev 18, 183–194. 27862851 10.1111/obr.12478 PMC5245104

[22] Smith LT (1999) Decolonizing Methodologies: Research and Indigenous Peoples. London: Zed Books.

[23] Cram F (2001) Rangahau Māori: Tona tika, tona pono – the validity and integrity of Māori research. In Research Ethics in Aotearoa, pp. 35–52 [ M Tolich , editor]. Auckland (NZ): Longman.

[24] Ministry of Health (2017) HISO 10001:2017 Ethnicity Data Protocols. Wellington (NZ): Ministry of Health.

[25] Braun V & Clarke V (2019) Reflecting on reflexive thematic analysis. Qual Res Sport Exerc Health 11, 589–597.

[26] Curtis E (2016) Indigenous positioning in health research: the importance of kaupapa māori theory-informed practice. AlterNative 12, 396–410.

[27] Airini DBC , Elana J , Odie L et al. (2009) Success for all: Improving Māori and Pasifika student success in degree-level studies. Auckland. https://cdn.auckland.ac.nz/assets/education/about/schools/crstie/docs/final-report-success-for-all-Dec09.pdf (accessed February 2020).

[28] Curtis ET , Wikaire E , Lualua-Aati T et al. (2012) Tātou Tātou/Success for all: Improving Māori Student Success. Wellington: Ako Aotearoa National Centre for Tertiary Teaching Excellence.

[29] World Health Organization (2016) Report of the Commission on Ending Childhood Obesity. Geneva (CH): World Health Organization.

[30] New Zealand Medical Association (2014) Tackling Obesity. https://assets-global.website-files.com/5db268b46d028bbc0fc0b537/5e26a5145f16d0d4bd430a54_Tackling%20obesity%20.pdf (accessed February 2020).

[31] Commission on Social Determinants of Health (2008) Closing the Gap in a Generation: Health Equity Through Action on the Social Determinants of Health. Final Report of the Commission on Social Determinants of Health. Geneva: World Health Organisation.

[32] Swinburn BA , Sacks G , Hall KD et al. (2011) The global obesity pandemic: shaped by global drivers and local environments. Lancet 378, 804–814. 21872749 10.1016/S0140-6736(11)60813-1

[33] Ministry of Health (2018) Neighbourhood deprivation. https://www.health.govt.nz/our-work/populations/maori-health/tatau-kahukura-maori-health-statistics/nga-awe-o-te-hauora-socioeconomic-determinants-health/neighbourhood-deprivation (accessed February 2020).

[34] Zambas SI & Wright J (2016) Impact of colonialism on Maori and Aboriginal healthcare access: a discussion paper. Contemp Nurse 52, 398–409. 27260472 10.1080/10376178.2016.1195238

[35] Armstrong SC & Skinner AC (2016) Defining “success” in childhood obesity interventions in primary care. Pediatrics 138, e20162497. 27621411 10.1542/peds.2016-2497

[36] Warbrick I , Came H , Dickson A et al. (2018) The shame of fat shaming in public health: moving past racism to embrace indigenous solutions. Public Health 176, 128–132. doi: 10.1016/j.puhe.2018.08.013. 30352699

[37] Guthold R , Stevens GA , Riley LM et al. (2020) Global trends in insufficient physical activity among adolescents: a pooled analysis of 298 population-based surveys with 1.6 million participants. Lancet Child Adolesc Health 4, 23–35. 31761562 10.1016/S2352-4642(19)30323-2 PMC6919336

