# Supplementary material for: Challenges of making healthy lifestyle changes for families in Aotearoa/New Zealand
Source: Public Health Nutr. 2020 Nov 6;24(7):1906–15. doi: 10.1017/S1368980020003699 (PMC8094428; doi:10.1017/S1368980020003699)
Supplement: Supplementary file 1 [file S1368980020003699sup.zip › S1368980020003699sup001.docx]

**COREQ (Consolidated criteria for reporting qualitative research)**

This checklist is intended to supplement the manuscript by providing further detail on methodology. The methodology has also been published previously.

**Domain 1: Research team and reflexivity**

*Personal characteristics*

1. Interviewers

Author 1 & Author 2 conducted the interviews.

2. Credentials and 3. Occupation

Author 1 (PhD) – Liggins Institute, University of Auckland

Author 2 – BHSc student, Te Reo Māori teacher (tertiary level) and interview facilitator, Liggins Institute, University of Auckland

Author 3 (MbChB, Dip Obs, FRACP) – Professor and paediatric endocrinologist, Liggins Insitute, University of Auckland and Auckland District Health Board

Author 4 (PhD) – Lecturer in Hauora Māori, Kōhatu – Centre for Hauora Māori, University of Otago

Author 5 (PhD, Dip Paeds, MBChB, FRACP, BSc [Psych]) – Senior Lecturer and paediatrician, Department of Paediatrics: Child and Youth Health, Faculty of Medical and Health Sciences, University of Auckland and Taranaki District Health Board

4. Gender & ethnicity

Author 1 – female, New Zealand European

Author 2 – female, Māori (Ngāti Mutunga, Ngāti Tama, Ngāti Rāhiri o Te Ati Awa me Ngai Tūhoe)

Author 3 – male, New Zealand European

Author 4 – female, Māori (Ngāti Toarangatira, Ngāti Koata me Ngā Ruahine)

Author 5 – female, New Zealand European

5. Experience and training

Author 1 had qualitative research training through the PhD (supervised by Authors 3-5). Author 2 had interview experience through her career. Author 3 is an experienced researcher in child health and endocrinology. Author 4 is an experienced qualitative researcher and has extensive experience in Māori health research. Author 5 is an experienced researcher in child health, especially childhood obesity.

*Relationship with participants*

6. Relationship established

All participants were recruited as described below, and some were already known to Author 2 through relationship networks.

7. Participant knowledge of the interviewer

The participants knew the reasons for conducting the research (detailed in the patient information and consent form), and participants were aware that the study would specifically ask about the factors that contributed to their decisions to engage or not engage, in order to improve the service. Participants were also aware that the interviewers were separate and distinct from the clinical service team.

8. Interviewer characteristics

Author 2 is a Māori researcher (of Ngāti Mutunga, Ngāti Tama, Ngāti Rāhiri o Te Ati Awa and Ngai Tūhoe descent) and Author 1 is New Zealand European. This mixed Indigenous – non-Indigenous partnership allowed us to connect and establish rapport with participants, depending on the interview participant and context. Author 2’s role as a parent had the advantage that familiarity with this stage of life helped her understand participants’ stories and ask exploratory questions.

**Domain 2: Study design**

*Theoretical framework*

9. Methodological orientation and Theory

The research approach of the original study was informed by Kaupapa Māori theory and Community-Up research principles. The approach was developed to minimise any perceived power imbalances between the interview team and the participants and make the interview experience as comfortable as possible. We used thematic analysis to analyse the interviews.

*Participant selection*

10. Sampling and 11. Method of approach

We identified eligible potential participants who fit the criteria, and then stratified these participants into groups by engagement level and ethnicity. We then randomised the list of potential participants in each group, and then contacted each participant one by one (stratified random sampling). Participants were recruited by telephone and interviewed in person over a six-month period from June to December 2018.

12. Sample size

For funding and resource reasons, we had a maximum total of 74 potential interviews with families. We conducted 64 interviews in total.

13. Non-participation

We approached 136 families, of which 53 were uncontactable, 7 agreed but were unable to be interviewed as they had moved out of the region, and 12 declined because they were not interested, were too busy with work, or did not remember the referral.

*Setting*

14. Setting of data collection

Participants were interviewed in person at their home, workplace, or at a local community child health centre. All participants chose where they would prefer to be interviewed.

15. Presence of non-participants

For most interviews, only the participant and interviewers were present, but there were occasionally other family members present, such as young children (<5 years).

16. Description of sample

Half the interviews were with families with Māori children. Most (80%) were solely with a female parent/caregiver (13 interviews included male parents and/or caregivers). 11 interviews involved two or more family members. 5 interviews included a child participant.

*Data collection*

17. Interview guide

A semi-structured interview framework was used and adjusted for relevance as each interview progressed. It was not pilot tested. The guide has been included as supplementary material.

18. Repeat interviews

Repeat interviews were not conducted, but participants were offered their transcripts for review after the interview.

19. Audio recording

Interviews were audiotaped digitally and transcribed by a medical typist.

20. Field notes

Field notes were made after each interview and kept as part of a reflexive notes.

21. Duration

Interview audio recordings ranged from nine minutes to 107 minutes (mean 31 minutes).

22. Data saturation

Data saturation was reached in each group of participants in each level of engagement.

23. Transcripts returned

Transcripts were anonymised and returned to the participant for checking, including deletions of portions if desired.

**Domain 3: analysis and findings**

*Data analysis*

24. Number of data coders

Eight transcripts were independently coded by Author 1 and Author 3 and discussed for consistency. After the coding matrix was constructed and consensus on codes reached, all transcripts were coded again by Author 1.

25. Description of the coding tree

A ‘mind map’ was used instead of a coding tree in order to better capture complexity and avoid an artificial hierarchy that did not adequately represent the inter-relationships between the themes, since themes could become more major or minor depending on the context.

26. Derivation of themes

Themes were derived from the data according to Braun and Clarke’s 2006 and 2019 method for reflexive thematic analysis.

27. Software

MAXQDA software was used to manage the data.

28. Participant checking

Participants did not provide feedback on the findings.

*Reporting*

29. Quotations presented

Participant quotations are presented in Table Two and throughout the manuscript. They are not identified by participant number.

30. Data and findings consistent

There was good consistency between data and findings, with the two interviewers working to discuss findings and the wider research team providing critique and challenging interpretations of data.

31. and 32. Clarity of major and minor themes

A distinction was made between ‘major’ and ‘minor’ themes with two key themes overlying the remaining six themes.

Reference: Tong A, Sainsbury P, Craig J. Consolidated criteria for reporting qualitative research (COREQ): a 32-item checklist for interviews and focus groups. Int J Qual Health Care 2007; 19, 349–357. doi: 10.1093/intqhc/mzm042

NB. This checklist has been amended to capture ethnicity and gender breakdown, to reflect the composition of the research team.
