# Supplementary material for: Challenges of making healthy lifestyle changes for families in Aotearoa/New Zealand
Source: Public Health Nutr. 2020 Nov 6;24(7):1906–15. doi: 10.1017/S1368980020003699 (PMC8094428; doi:10.1017/S1368980020003699)
Supplement: Supplementary file 1 [file S1368980020003699sup.zip › S1368980020003699sup002.docx]

**SUPPLEMENTARY MATERIAL: Interview schedule**

*NB. Interviews were semi-structured and this schedule is simply a guide to the line of interview questioning. Not all questions were asked if not appropriate. Sections marked with an asterisk (*) were not asked of participants who declined involvement in the programme.*

**Referral & initial thoughts**

- Who referred you to Whānau Pakari?
- Do you remember how they told you about the programme? *(if not self-referral)*
- Did they talk with you and your child?
- How did you feel about being referred? How did you talk to your child/children about the programme and referral?
- Why did you decide to accept the referral?
- How did you feel about starting the programme?
- Which part of the programme (if any) were you more interested in, or seemed more relevant for you and/or your family?
- Were you worried about what other people might think about you and your family attending Whānau Pakari?

**Overall experience ***

- Which parts of the programme were the most helpful?
- Which parts of the programme did you find were more difficult?
- Did Whānau Pakari meet your needs in supporting you to make healthy lifestyle changes as a family? Why/why not?
- Did Whānau Pakari meet your expectations in terms of what you thought the team would provide? How did/didn’t they do this?
- Would you recommend Whānau Pakari to other families and why/why not?

**Barriers and facilitators ***

- What were the things that helped you to or made you want to continue to attend Whānau Pakari sessions, if any?
- What were the things that made it hard for you to continue to attend Whānau Pakari, if any?
- Did you experience any travel barriers to get to the sessions? E.g. location, access to car, parking, petrol, WOF, rego
- The Healthy Lifestyles Coordinator came to you for the assessments. What were the good things about a home visit? (If any) What were the negative things? (If any)
- Do you prefer home-based assessments rather than coming to the hospital/clinic?
- Were you able to involve the rest of your children and family in the programme sessions.
- How much of a priority was Whānau Pakari in relation to your other demands?
- How did other competing demands, obligations or choices impact on your decision to attend Whānau Pakari?
- Can you please describe how Whānau Pakari was (or wasn’t) suitable for families like yours?
- Was Whānau Pakari family-friendly, inviting, comfortable? What could be done to improve this?
- Can you please tell me how costs may or may not have been barriers to participation in Whānau Pakari? How?
- Can you tell me about any other barriers to participation in Whānau Pakari that you experienced?
- Can you think of things that might have motivated you or your whānau to participate (or things that kept you from participating)?

**Beliefs and feelings around healthy lifestyle programmes**

- What do you think about healthy lifestyle programmes in general?

**Previous experiences with health system**

- In general, can you describe what your experiences with the health system have been like prior to the Whānau Pakari programme?
- Have you ever felt that you or your family have been treated unfairly in the health system? If yes, why do you think that this happened?
- In your experiences with the health system, have you ever experienced discrimination? If yes, can you tell me a bit more about this? Why do you think this happened?
- Have any of you or your family’s previous experiences with the health system influenced your decision to attend Whānau Pakari? If yes, how?
- Have you had any previous negative experiences with health providers that made you choose not to attend Whānau Pakari sessions?

**Previous experiences with societal stigma**

- In your day-to-day life, have you ever been treated unfairly or discriminated against? If yes, can you tell me a bit more about this? Why do you think this happened?
- Have you ever witnessed or heard about other members of your whānau being treated unfairly or discriminated against for any reason? If yes, can you tell me a bit more about this?
- Do you think any of these experiences influenced your decisions or ability to attend, engage, participate or continue participation in Whānau Pakari?

**Follow-up prompt guidelines**

Prompts are to keep the momentum of the interview by being positive, affirming answers and using active listening without being leading. Prompts/probes are to facilitate elaboration, continuation, clarification, attention and completion.

- Prompts included:
  - Non-verbal nods
  - Affirming sounds e.g. Mmmm
  - Agreement e.g. yes, that’s interesting
  - Questions e.g. “what did you mean by that?”, repeat the last word the participant said as a question to prompt more explanation.
  - To get back on track: “Could I ask you about something you said before…”
